# Efficacy and safety of alfaxalone compared to propofol in canine refractory status epilepticus: a pilot study

**DOI:** 10.3389/fvets.2024.1383439

**Published:** 2024-07-08

**Authors:** Tania Al Kafaji, Andrea Corda, Marios Charalambous, Elsa Murgia, Ilaria Tartari, Mariangela Puci, Pasquale Debidda, Antonella Gallucci

**Affiliations:** ^1^Veterinary Neurological Center “La Fenice”, Selargius, Italy; ^2^Department of Veterinary Medicine, Veterinary Teaching Hospital, University of Sassari, Sassari, Italy; ^3^Small Animal Clinic, Blaise Veterinary Referral Hospital, Birmingham, United Kingdom; ^4^Department of Veterinary Medicine, University of Hannover, Hannover, Germany; ^5^Clinical Epidemiology and Medical Statistic Unit, Department of Medicine, Surgery and Pharmacy, University of Sassari, Sassari, Italy

**Keywords:** status epilepticus, alfaxalone, propofol, dogs, seizures

## Abstract

**Introduction:**

Refractory status epilepticus (RSE) is defined as seizure activity that is minimally responsive to first- or second-line antiseizure medications. Constant rate infusion (CRI) intravenous propofol (PPF) is commonly used to treat RSE in dogs and cats. The antiseizure activity of alfaxalone (ALF) in RSE has been demonstrated in various experimental studies. This study compared the clinical efficacy and safety of intramuscular administration followed by CRI infusion of ALF with intravenous administration followed by CRI infusion of PPF to treat canine RSE.

**Materials and methods:**

This was a multicenter, prospective, randomized clinical trial of client-owned dogs referred for status epilepticus that did not respond to first- and second-line drugs. Animals with suspected or confirmed idiopathic or structural epilepsy were included. The dogs were randomly assigned to either the PPF or ALF treatment groups and each group received drug CRI infusions for 6 h. Drug dosages were progressively reduced by 25% every hour from the third hour until suspension after 6 h. Patients were classified as responders or non-responders based on the relapse of epileptic seizures during the 24 h therapy infusion or within 24 h of drug suspension. Univariate statistical analyses were performed.

**Results:**

Twenty dogs were enrolled in the study. Ten (10/20) dogs were randomly allocated to the PPF group and 10 (10/20) to the ALF group. Successful outcomes were obtained in six (6/10) patients in the PPF group and five (5/10) patients in the ALF group. Adverse effects were recorded in six (6/10) and three (3/10) animals in the PPF and ALF groups, respectively. No statistically significant differences in outcomes or the presence of adverse effects were observed between the groups.

**Discussion:**

The results of this preliminary study suggest that ALF can be considered a valid and safe alternative to PPF for the treatment of RSE in dogs, with the additional advantage of intramuscular administration. However, caution should be exercised when using these drugs to provide airway and hemodynamic support.

## 1 Introduction

Status epilepticus (SE) is defined as prolonged seizure activity lasting >5 min or two or more seizures in the absence of complete recovery of consciousness between occurrences (1, 2). Refractory SE (RSE) is an epileptic state that is not responsive to first- or second-line antiseizure medication (ASM) (2, 3). SE is a life-threatening emergency requiring prompt therapeutic intervention, with a reported mortality rate ranging from 25.3 to 38.5% (1). According to the guidelines (1, 4–6), benzodiazepines (BZDs) are the first-line anticonvulsant therapies recommended to enhance γ-aminobutyric acid (GABA)ergic transmission (7). If BZDs fail to cease seizure activity and/or SE has already progressed beyond the impending stage, a polytherapeutic approach is indicated. Initial administration of phenobarbital (PB) and/or levetiracetam (LEV) as the most common second-line drugs is recommended, followed by third-line drugs, including general anesthetic agents. Ketamine (+/− dexmedetomidine) is considered the third-line approach; however, ketamine has also been proposed after BZDs failure (1, 4). An intravenous (IV) bolus of propofol (PPF) followed by constant rate infusion (CRI) is also recommended as a third-line medication (1, 4). Pentobarbital and inhalant anesthetic drugs are usually third-line medications that are recommended following the failure of PPF (1, 4, 5).

Previous studies have shown a progressive decrease in the efficacy of BZDs and PB with increasing time from the onset of SE (specifically after 30 min) (1). Internalization of GABA-A receptors, increased expression of drug efflux transporters, and overexpression of N-methyl-D-aspartate receptors are the main mechanisms implicated in the development of ASM resistance during SE (1, 5–8). Antiepileptic drugs that bind to different receptor sites or have other mechanisms of action have been proposed for RSE (1, 4).

PPF is an anesthetic agent commonly used in the treatment of RSE in dogs and cats (6, 9–11), acting as a GABA-A agonist and N-methyl-D-aspartate receptor antagonist. CRI is indicated because of its short elimination time (6), although specific indications for the optimal CRI duration are lacking (9). Cardiovascular and respiratory depressions have potential adverse effects with PPF use. In addition, its use has been associated with Heinz body formation in cats (12) and oxidative-induced anemia in dogs (13). PPF CRI has also been associated in a dog with onset of rhabdomyolysis, myoglobinuria, cardiac arrythmia, alteration of liver enzymes and methemoglobinemia (14). Furthermore, in humans, PPF infusion syndrome was described in RSE during PPF CRI infusion lasting 8–426 h (15). An open-label, nonrandomized canine clinical trial revealed that a shorter PPF CRI infusion was not associated with worse outcomes compared with a longer CRI duration (9).

Alfaxalone (ALF) is a neurosteroid general anesthetic drug that acts on synaptic and extrasynaptic GABA-A receptor subunits (16). It promotes neuronal hyperpolarization by increasing the influx of intracellular chloride ions (16). Besides the IV administration route, intramuscular (IM) injection may be a safe and reliable way to achieve an anesthetic effect in dogs and cats (17, 18). The antiepileptogenic activity and potential beneficial role of neurosteroids in RSE have been demonstrated in various experimental studies (18–22). In fact, extrasynaptic GABA-A receptor subunits play a crucial role and are considered an important target for the treatment of benzodiazepine-resistant SE (22). ALF has been characterized with excellent tissue distribution, including in the central nervous system, rapid onset of action, an a half-life of up to 30 min in dogs and a good safety profile (23). Hypoventilation and apnea are the most prevalent dose-dependent side effects described (24). Nevertheless minimal occurrences of cardiorespiratory depression have been reported with common therapeutic doses (24). Its pharmacokinetic profile makes it possible to use it both in repeated boluses and in continuous infusions (without clinically relevant accumulation) with fast and predictable awakenings (24–29).

In veterinary medicine, data on the use of ALF in the treatment of SE is lacking. Its action on extrasynaptic GABA-A receptors during RSE could represent a potential advantage for evaluating ALF as an alternative method to the other currently drugs. In addition, the unique ability for ALF to be administered either IM or IV makes it crucial for patients who do not have immediate venous access because they are experiencing severe and non-improving tonic-clonic epileptic seizures.

This prospective study evaluated the efficacy of ALF administered first by IM followed by 6 h of IV CRI infusion in controlling seizure activity during canine RSE. This was compared to treatment with IV PPF followed by 6 h of CRI infusion. We also assessed the safety and potential adverse effects of PPF.

## 2 Materials and methods

### 2.1 Study participants

This study was a prospective, randomized, parallel-group controlled clinical trial conducted on client-owned dogs. The study design was approved by the ethical committees of the institutions that enrolled the cases. Informed consent was obtained from all owners. All dogs included in the present study were referred for SE that manifested as generalized or focal seizures. SE was defined as seizures lasting ≥5 min or ≥2 seizures without complete recovery of consciousness between episodes (2). Dogs were enrolled in the trial if they experienced prolonged SE for at least 30 min after the administration of first- and second-line drugs. According to the guidelines (4), dogs were initially treated with two or three repeated boluses of BZDs administered by intra-nasal, IV, or IM route (midazolam 0.1–0.3 mg/kg or diazepam 0.5–2 mg/kg), followed by a CRI of midazolam (0.1–0.3 mg/kg/h) or diazepam (0.1–0.5 mg/kg/h). The second-line drugs were IM administered PB and/or IV administered LEV. Dogs were randomly assigned to either the PPF or ALF treatment group by drawing lots. All extractions were blinded and performed by an unbiased medical staff member not involved in the study. Only dogs with a suspected diagnosis of idiopathic epilepsy (IE) or structural epilepsy (StE) were included, whereas those with suspected reactive seizures were excluded. Patients allocated to the PPF group were treated with an initial IV bolus at a dosage ranging from 1–4 mg/kg followed by a CRI of IV PPF (0.1–0.4 mg/kg/min) for 6 h. The drug dosage was progressively reduced by 25% every hour, starting from the third hour until suspension after 6 h. In the ALF group, a first bolus of ALF at a dosage of 2–3 mg/kg was administered by IM injection, followed by a CRI of IV ALF (0.10–0.15 mg/kg/min) for 6 h. The maximum volume of the ALF IM injection was 0.25 mL/kg/site. If necessary, the dosing solution was injected into two separate sites on the dorsal lumbar muscle. The CRI of IV ALF was started immediately after the placement of an IV catheter following the initial IM injection. Therapy was then tapered by 25% every hour, starting from the third hour, for a total administration time of 6 h. Regarding the choice of ALF dosages, we selected the minimum IM dose needed to achieve enough sedation in dogs, basing on previous studies (17). For IV CRI, we referred to the suggested drug dosages for anesthesia maintenance (28), adjusting each treatment protocol in relation to individual dog’s responses. All patients were hospitalized in the intensive care unit and closely monitored during drug infusion. Continuous evaluation of vital parameters, such as oscillometric blood pressure measurements (petMAP graphic II, Vetefarma, Italy), oxygen saturation (monitored using a pulse oximeter), and electrocardiogram, were performed. Animals were classified as responders (R+) or non-responders (R−) basing on reoccurrence of epileptic seizures during therapy infusion or within 24 h from the time of drug suspension. “Non-responder” patients were treated with additional antiseizure and anesthetic medications, according to the personal choice of the medical team. Seizure relapse was the only criterion used for classifying the outcome, although we considered also progressive improvement in dog’s mental status and neurological condition in the overall clinical evaluation.

The “seizure cessation” time was defined as the interval of time between administration of IV PPF or IM ALF bolus and resolution of SE (30). “Seizure relapse” time was defined as the interval of time between cessation of SE and the reappearance of seizures.

Signalment, anamnesis, physical examination findings, laboratory test results, suspected seizure etiology, duration of epileptic seizures before presentation, and chronic ASM were recorded. A suspected diagnosis of IE was made based on the following parameters: (1) the patient’s age at the time of seizure onset was between 6 months and 6 years; (2) normal inter-ictal neurological examination performed by an European College of Veterinary Neurology Diplomate (XX) or Resident (XX); (3) unremarkable comprehensive blood exams containing complete blood cell count, serum biochemistry, and fasted ammonia, and (4) a normal brain magnetic resonance image (MRI) and cerebrospinal fluid (CSF) analysis or a history of seizures longer than 1 year. StE was suspected in patients older than 6 years at the beginning of the seizures and/or with an abnormal interictal neurological examination. If available, MRI, CSF results, and electroencephalography (EEG) findings were included. Further information regarding potential side effects and deaths during hospitalization was also recorded.

### 2.2 Statistical analysis

Differences in the collected variables, in terms of signalment, seizures features, final diagnosis, outcome and potential side effects, were compared between the PPF and ALF treatment groups, as well as between R+ and R− subjects. In the latter case, the variables were compared for the entire population and individual groups (PPF and ALF). Qualitative variables were described using absolute and relative frequencies, whereas quantitative variables were summarized as medians and interquartile ranges (IQR). Differences between study groups were assessed using the chi-squared and Fisher’s exact tests for qualitative variables and the Mann–Whitney *U* test for quantitative variables. Statistical significance was set at *p* < 0.05. Statistical analyses were performed using STATA17 software (Stata Corp, College Station, TX, United States).

## 3 Results

### 3.1 General population

Twenty dogs were enrolled in the study. Ten dogs (10/20) were randomly allocated in the PPF group, and 10 (10/20) in the ALF group. There were 13 (13/20) males, of which four (4/13) were neutered, and seven (7/20) females, of which six (6/7) were spayed. The study population included two Pinschers (2/20), two Yorkshire Terriers (2/20), two French Bulldogs (2/20), two English Bulldogs (2/20), two mixed-breed dogs (2/20), and one (1/20) of each of the following breeds: Australian shepherd, Saint Bernard, Labrador Retriever, Beagle, Pug, American Cocker, Chihuahua, Dachshund, Poodle, and Bull Terrier. Demographic data, such as sex, body weight, and age, are shown in Table 1.

**Table 1:** Demographic data of the study population.

Variable	Dogs (*n* = 20)
Sex, male, *n* (%)	13 (65)
Sex, female, *n* (%)	7 (35)
Weight, kg, median (IQR)	12.7 (5.75–25.3)
Age years, median (IQR)	6.2 (2.25–8)

*n*, number of dogs; IQR, interquartile range.

The median IQR time elapse between the onset of SE and administration of the first-line drug was 40 (IQR 30–120) minutes (range 10–720 min). Eleven dogs (11/20) presented with cluster seizures before the appearance, and 11 (11/20) had chronic ASM. Sixteen dogs (16/20) underwent advanced diagnostic imaging, 15 (15/20) underwent MRI and one (1/20) underwent computed tomography. Fifteen dogs that underwent MRI also underwent CSF examination and five patients (5/20) underwent EEG. IE was diagnosed in nine (9/20) dogs (all with MRI and CSF examination), while 11 dogs (11/20) were presumed to have StE. Five (5/20) dogs were suspected of having neoplastic disease (three cases and one case underwent MRI and CT scan, respectively), three (3/20) of having a vascular accident, and three (3/20) of inflammatory disorders, such as meningoencephalitis of unknown origin, were diagnosed with MRI and CSF examination.

Overall, a successful outcome was obtained in 11 dogs (11/20) classified as R+, and nine (9/20) patients experienced a relapse of one or more epileptic seizures during the following 24 h (Table 2). None of the dogs experienced further SE during hospitalization. Nine dogs (9/20) had side effects during hospitalization: apnea in three (3/20), of which two (2/20) required assisted ventilation; hypothermia in two (2/20); hypotension in two (2/20); hypertension in one (1/20); and hyperthermia in one (1/20). Overall, no significant differences were found between the R+ and R− groups.

**Table 2:** Outcome and side effects in comparison between the groups.

Variables		PPF group (*n* = 10)	ALF group (*n* = 10)	*p*-value
Responders, *n* (%)		6 (60)	5 (50)	1.00
24 h relapse, *n* (%)		4 (40)	5 (50)	1.00
Side effects, *n* (%)		6 (60)	3 (30)	0.37
Side effects	Hypotension, *n* (%)	2 (20)	0 (0.0)	0.47
	Hypertension, *n* (%)	1 (10)	0 (0.0)	1.00
	Hyperthermia, *n* (%)	1 (10)	0 (0.0)	1.00
	Hypothermia, *n* (%)	2 (20)	0 (0.0)	0.47
	Apnea, *n* (%)	0 (0.0)	3 (30)	0.09

PPF, propofol; ALF, alfaxalone; *n*, number of dogs.

Body weight was the only demographic that was significantly different between the PPF and ALF treatment groups, which was significantly higher in the PPF group (Table 3).

**Table 3:** Study population and comparison between groups.

Variables		PPF group (*n* = 10)	ALF group (*n* = 10)	*p*-value
Sex, male, *n* (%)		8 (80)	5 (50)	0.35
Sex, female, *n* (%)		2 (20)	5 (50)	
Reproductive state neutered, *n* (%)		4 (40)	6 (60)	0.65
Body weight, kg, median (IQR)		25.3 (14–29)	6 (4.7–12)	0.003^*^
Age, years, median (IQR)		3.3 (2–6.8)	8 (2.5–11)	0.12
SE onset, minutes, median (IQR)		37.5 (30–40)	120 (30–180)	0.25
CS presence, *n* (%)		6 (60)	5 (50)	1.00
MRI and CSF examination, *n* (%)		6 (60)	9 (90)	0.30
EEG examination, *n* (%)		1 (10)	4 (40)	0.30
Diagnosis, IE, *n* (%)		6 (60)	3 (30)	0.37
Diagnosis StE, *n* (%)		4 (40)	7 (70)	
StE	Neoplasia, *n* (%)	2 (20)	3 (30)	1.00
	Vascular, *n* (%)	1 (10)	2 (20)	1.00
	Meningoencephalitis of unknown origin, (%)	1 (10)	2 (20)	1.00
Chronic ASM administration, *n* (%)		6 (60)	5 (50)	1.00

PPF, propofol; ALF, alfaxalone; *n*, number of dogs; IQR, interquartile range; SE, status epilepticus; CS, cluster seizures; MRI, magnetic resonance imaging; CSF, cerebrospinal fluid; EEG, electroencephalography; IE, idiopathic epilepsy; StE, structural epilepsy; ASM, anti-seizures medication.

### 3.2 PPF group

Signalment data are summarized in Table 1.

A successful outcome was obtained in six cases (6/10) that were classified as R+, and a relapse of one or more epileptic seizures during the following 24 h was observed in four cases (4/10). None of the dogs experienced further SE during hospitalization. Adverse effects were noted in six cases (6/10) and were characterized by hypothermia (2/6), hypotension (2/6), hypertension (1/6), and hyperthermia (1/6). All of these conditions required medical treatment and resolved during CRI administration.

### 3.3 ALF Group

Signalment data are summarized in Table 1.

A successful outcome was obtained in five cases (5/10) that were classified as R+, and a relapse of one or more epileptic seizures during the following 24 h was observed in five cases (5/10). Adverse effects were noted in three cases (3/10). In all three cases, the dogs experienced apnea at the time of IV bolus administration, with 2 dogs requiring assisted ventilation for 10 and 16 min.

## 4 Discussion

In this study, no significant differences were noted between the PPF and ALF treatment groups in terms of their efficacy for RSE. Although this study was conducted in only 20 dogs and, given the small sample size, the data collected may not adequately represent the larger population and should be interpreted with caution, we can assume that ALF could be considered a valid alternative to PPF in controlling SE in dogs.

To the best of our knowledge, this is the first study to evaluate the use of ALF in patients with canine RSE. The rationale of this study was to consider anesthetic agents other than PPF for controlling seizure activity during the BZD-resistant phase of SE. Neuroactive steroid-positive allosteric modulators of GABA-A receptors enhance both synaptic and extrasynaptic GABA-A receptors and may be effective in the treatment of refractory SE (4, 31, 32). In an experimental study conducted in mice, dose-dependent IV and IM allopregnanolone seizure protection was observed, with a rapid protective effect that declined within 15–60 min (depending on dose and administration route) (22). During the induction of BZD-resistant SE by the administration of kainic acid, a potent neuroexcitatory amino acid, mice treated with either diazepam or allopregnanolone achieved seizure freedom at the early and late observation times. However, when the treatments were administered 40 min after kainic acid, which is at a more advanced stage of SE, only 25% of the animals in the diazepam group were seizure-free at the late observation time, whereas all animals that had received allopregnanolone were seizure-free at this time point (22). These findings enhance the potential advantage of neurosteroids administration in more advanced stages of SE during which the function of BZD declines and support the potential utilization of ALF as an alternative drug for treating RSE. In our cohort, ALF led to interruption of seizure activity in half of dogs previously treated with BZDs, supporting the findings of earlier experimental studies (22).

The results of this preliminary study showed that the PPF group had a higher percentage of outcome success and a lower percentage of seizure relapse during the following 24 h than the ALF group (60% vs. 50 and 40% vs. 50%, respectively). Although the difference was not statistically significant, a possible explanation for this may be that more dogs with IE were included in the PPF group, which could represent a potential bias positively affecting the outcome. In fact, IE may be associated a better outcome instead StE (33). Although our results showed no significant differences between R+ and R− cases with regard to the etiology of SE, studies with large canine populations are needed to investigate the influence of SE etiology on outcomes. StE may be associated with worse outcomes, similar to neoplasia. This study found that with the exception of body weight, none of the collected variables differed between the PPF and ALF treatment groups. A possible explanation for this result might be that in the PPF group, a 60 kg Saint Bernard dog was included, which may have influenced the median body weight value in such a small sample size. Another important finding was that no variable differed significantly between R+ and R− cases when considering the entire population and even when considering individual groups. However, with a small sample size, caution must be exercised, as the findings might not be generalizable to the general population.

This study showed a good response to treatment in both groups, similar to that reported for longer PPF CRI protocols (34). A significant reduction in ALF and PPF CRI durations was chosen to evaluate the efficacy of shorter protocols, cost reduction, and potential adverse effects. In humans, high doses (>4 mg/kg/h) or prolonged CRI infusion (>48 h) of PPF can lead to PPF syndrome, consisting of metabolic acidosis, rhabdomyolysis, hyperkalemia, leukemia, renal failure, hepatomegaly, and cardiovascular collapse (35). This seems to be due to impaired mitochondrial activity due to the use of free fatty acids, which results in a mismatch between energy needs and use (36). In veterinary patients, such a syndrome has not been documented, but it can potentially occur, especially in patients maintained on a long PPF CRI (7). A successful outcome (R+) was observed in almost half of the patients despite the nature of the epilepsy. We can speculate that in some cases, a shorter anesthetic protocol may be used, can then subsequently be prolonged in non-responsive patients. In addition, a recent study in dogs reported that seizure relapse occurred within 7 h from the hospital admission in half of the population (37). In our study, seizure recurrence during or within 6 h from the end of drug infusion was selected as a criterion to classify dogs as responders or not. Although this, we cannot exclude that a longer anesthetic protocol could have led to different results in our population study. The use of EEG may be useful for monitoring early epileptic burst suppression and good anesthetic protocol management. In addition, EEG monitoring could be an important tool to adjust the duration of therapeutic protocols in relation to individual patients.

A further aim was to assess the safety and potential side effects of ALF compared with those of PPF. Even though only 30% of cases showed adverse effects in the ALF, two dogs required mechanical ventilation for apnea. This is a crucial point because this has also been described for PPF CRI infusion. Therefore, it is highly recommended that these medications are administrated with airway support in the form of endotracheal intubation, assessment of respiratory parameters in spontaneous breathing patients and the additional use of mechanical ventilation in case of hypercapnia and/or oxygen desaturation. Also hemodynamic support might be ensured with clinical and instrumental evaluation of perfusion parameters. Experimental studies investigating the cardiorespiratory and anesthetic effects of ALF in dogs and cats have suggested minimal cardiorespiratory depression, excellent safety margins, and dose-dependent anesthetic properties (38). However, the administration of higher doses of ALF (6 and 20 mg/kg IV) increased the heart rate and produced dose-dependent decreases in arterial blood pressure and mean pulmonary arterial pressure without changes in cardiac output and mean right atrial pressure (24). The same experimental study also showed that the administration of ALF produced a dose-dependent decrease in the respiratory rate. The duration of apnea was directly related to the ALF dose and was more frequent in dogs administered 6 and 20 mg/kg than in those administered the 2 mg/kg dose. Based on these data, it would be optimal to use the lowest possible dose of ALF, ideally achieved using EEG monitoring, to control seizure activity. Although muscle tremors were also described in dogs after IM ALF administration (17), they have not been observed in our population study. Muscle twitching could be confused with persistence of SE and, in these cases, the use of an EEG exam could be an important tool for differentiate them.

The preliminary results of our study suggest that ALF could be considered a valid alternative to PPF for controlling SE in dogs, and this could represent an interesting opportunity for veterinarians, particularly in patients with difficult IV access. In fact, in an experimental study, the mean time needed to achieve lateral recumbency in dogs after ALF IM injection ranged from 167 to 232 s, depending on the dosage (17). This time could be helpful for the placement of an IV catheter and to take advantage in those difficult situations with patients hardly to manage. ALF can be administered through several parenteral routes and does not cause tissue irritation upon extravasation, thus offering a distinct advantage over PPF (39). Injection of a large IM volume of ALF may induce discomfort (e.g., vocalizing or struggling). In our study, a low volume was used per injection site. No swelling or changes in the skin were observed around the site of the IM injection that would suggest the presence of discomfort. The use of a larger volume of ALF, which is necessary in large-breed dogs, may represent a limitation for awake patients. However, an experimental study of six dogs receiving three IM doses each of ALF at increasing dose rates every other day, did not find a significant difference in the incidence of discomfort during 5.1 +/−0.4 mL IM administration (17).

The main limitation of the present study was the small number of recruited dogs, which may have affected the statistical power and limited the inference of the results. Other limitations could be represented by variability in drug dosages between different institutions or patients, even if they were within the reference ranges indicated for each drug. Diagnosis of structural epilepsy, as we discussed before, could have affected the results leading to a worse prognosis in comparison to an idiopathic seizures etiology. In addition, dogs were selected based also on agreement and financial sources of the owners and this could have led to potential bias. Further studies with larger population are necessary to better evaluate the efficacy of ALF in controlling seizure activity, considering a more prolonged CRI duration, standardized treatment protocols and longer follow-up periods. They may be useful to also investigate the effect of independent variables, such as epilepsy etiology, on outcome success in dogs with SE.

## Data availability statement

The datasets presented in this study can be found in online repositories. The names of the repository/repositories and accession number(s) can be found in the article/supplementary material.

## Ethics statement

The animal studies were approved by Organismo Preposto al Benessere e alla Sperimentazione Animale (OPBSA), University of Sassari (Italy). The studies were conducted in accordance with the local legislation and institutional requirements. Written informed consent was obtained from the owners for the participation of their animals in this study.

## Author contributions

TA: Conceptualization, Writing – original draft, Writing – review & editing. AC: Data curation, Formal analysis, Writing – review & editing, Software. MC: Conceptualization, Writing – review & editing. EM: Conceptualization, Methodology, Supervision, Writing – review & editing. IT: Methodology, Writing – review & editing. MP: Formal analysis, Writing – review & editing. PD: Conceptualization, Writing – review & editing. AG: Conceptualization, Methodology, Project administration, Supervision, Writing – original draft, Writing – review & editing.
